# Hemostatic potential of recombinant von Willebrand factor and standard or pegylated extended half-life recombinant factor VIII on thrombus formation under high shear flow

**DOI:** 10.1186/s12959-023-00569-1

**Published:** 2023-12-08

**Authors:** Hiroaki Yaoi, Yasuaki Shida, Kenichi Ogiwara, Keiji Nogami

**Affiliations:** 1 Department of Pediatrics, Nara Medical University, 840 Shijo-cho, Kashihara, Nara 634-8522 Japan; 2 Department of Pediatrics, Nara City Hospital, Nara, Japan

**Keywords:** Von Willebrand disease, Von Willebrand factor, Factor VIII, Shear stress, Thrombus formation

## Abstract

**Background:**

Von Willebrand factor (VWF) and factor VIII (FVIII) complex play a pivotal role in hemostasis. A deficiency or defect of VWF causes von Willebrand disease (VWD). Recombinant (r)VWF product has proved to be effective for hemostatic treatment of VWD, but limited information is available on their role in moderating thrombus formation under flow condition. We aimed to assess thrombus formation in the presence of rVWF combined with rFVIII or pegylated-extended half-life rFVIII (peg-EHL-rFVIII) in VWD whole blood under high shear flow.

**Methods:**

Perfusion chamber experiments under high shear (2,500 s^− 1^) combined with immunostaining were performed using patient’s whole blood with type 1 VWD, mixed with rVWF (Vonvendi^®^; 1.6 IU/mL), rFVIII or peg-EHL-rFVIII (Advate^®^ or Adynovate^®^; 1.0 IU/mL), or both. Similar experiments were also conducted with clinical medical devices (T-TAS^®^).

**Results:**

The addition of rFVIII did not augment thrombus formation assessed by surface coverage (SC) and thrombus height (TH), whereas rVWF enhanced these parameters (SC 19.1 ± 1.1% vs. 30.1 ± 4.1%, TH 2.2 ± 0.14 μm vs. 3.6 ± 0.40 μm, respectively). The co-presence of rVWF/rFVIII was comparable to plasma-derived VWF/FVIII (Confact^®^, VWF:FVIII ratio = 1.6:1.0) for increasing thrombogenicity in SC (32.5 ± 4.3% vs. 38.7 ± 5.5%) and in TH (5.0 ± 0.60 μm vs. 5.5 ± 0.64 μm), respectively. The pre-incubation time with rVWF and rFVIII appeared to have a little effect on the size of thrombus. Peg-EHL-rFVIII mediated thrombus formation to similar extent as rFVIII in the co-presence of rVWF. Similar results were obtained even with T-TAS. Immunostaining demonstrated that rFVIII and peg-EHL-rFVIII were similarly co-localized with rVWF in formed thrombi, indicating that pegylation did not interfere with molecular complexes.

**Conclusion:**

The effects of high-level rVWF and peg-EHL-rFVIII on thrombus formation were comparable to conventional therapeutic products in a patient’s whole blood with VWD under high shear flow.

## Introduction

Von Willebrand factor (VWF) plays a role in primary hemostatic mechanism associated with hemorrhage and thrombosis [1]. Under high shear stress conditions, VWF mediates the tethering of platelets to injured blood vessels by bridging the platelet-receptor glycoprotein (GP)Ib and subendothelial collagen [1]. Platelet adhesion on the damaged surfaces results in platelet activation and conformational changes of the GPIIb/IIIa receptor that binds to VWF and fibrinogen, thus promoting further platelet adhesion and aggregation at the same site [2]. VWF forms a complex with factor VIII (FVIII), protecting FVIII from proteolysis by serine proteases such as activated protein C and/or intracellular uptake [3]. Therefore, a deficiency or defect of VWF results in a rapid reduction in FVIII activity (FVIII:C) [2].

Von Willebrand disease (VWD) is the most common congenital bleeding disorder, and is caused by a defect or deficiency of VWF in circulating blood. VWD is classified into three categories, characterized by a quantitative deficiency of VWF (type 1), a qualitative abnormality of VWF (type 2), and undetectable VWF levels (type 3) [4]. Replacement therapy with plasma-derived (pd-)VWF/FVIII concentrate is the most reliable hemostatic treatment for all types of VWD [4]. Some drawbacks are evident, however. Plasma-derived materials pose a potential risk of viral infection that may not be completely eliminated [5]. In addition, the ratios of VWF and FVIII in individual products are variable depending on the plasma source, and can be difficult to maintain within an optimal hemostatic range [6]. Moreover, the amounts of the high molecular weight (HMW-)VWF multimer component, essential for platelet adhesion, may vary due to proteolysis [7].

Recombinant VWF product(s) (rVWF) have been more recently developed, and have demonstrated a slightly longer half-life (19.6–21.9 h) compared to plasma-derived VWF (13–16 h). In addition, clinical studies of rVWF demonstrated excellent stabilization of endogenous FVIII [8, 9]. In those phase 1 and phase 3 trials, initial on-demand treatment with rVWF together with recombinant FVIII product(s) (rFVIII), and subsequent therapy with rVWF alone was effective for a variety of bleeding symptoms as long as targeted FVIII:C levels were maintained. Furthermore, rVWF is now widely available for clinical practice in many countries, and has proved to be effective for hemostatic treatment of patients with VWD.

rVWF comprises intact, ultra-large (UL) and HMW-VWF multimers, and is not affected by ADAMTS13 (a disintegrin-like metalloprotease domain with thrombospondin type 1 motifs 13) during the manufacturing process. Infused UL-VWF is rapidly proteolyzed by endogenous ADAMTS13, however, possibly contributing the absence of thrombotic side effect. Limited information is available, however, on the functional properties of rVWF related to thrombus formation under physiologic flow conditions, and little data have been described on hemostatic mechanisms induced by the concomitant use of rVWF with standard rFVIII or pegylated extended-half-life rFVIII product(s) (peg-EHL-rFVIII) [10] in clinical practice. The present study was designed to examine the hemostatic potential of rVWF and rFVIII or peg-EHL-rFVIII in whole blood from patients with VWD using an ex vivo flow chamber under high shear flow.

## Materials and methods

This study was approved by the Medical Research Ethics Committee of Nara Medical University, and blood samples from a patient with type 1 VWD were collected after informed consent following the University ethical guidelines.

***Reagents—***Standard rFVIII (Advate^®^), peg-EHL-rFVIII (Adynovate^®^), and rVWF (Vonvendi^®^) were obtained from Shire Japan Limited (Tokyo, Japan), and plasma-derived VWF/FVIII concentrate (Confact^®^; VWF:FVIII ratio; 1.6:1.0; available back then) was purchased from KAKETSUKEN (Kumamoto, Japan). Collagen I/III (MP Biomedicals, Santa Ana, CA), recombinant hirudin (Enzo Life Science, Farmingdale, NY), anti-VWF antibody (DAKO, Santa Clara, CA), Cytoperm/Cytofix (BD, Franklin Lakes, NJ), phalloidin-Alexa 488, Alexa 568 and 647 labeling kit (Molecular Probes, Eugene, OR), were purchased from the indicated vendors. An anti-FVIII C2 polyclonal antibody (polyAb) was obtained from a severe hemophilia A patient with inhibitor was used for immunostaining as previously described [11, 12].

***Perfusion chamber experiments*–**Perfusion experiments were performed as previously described [13–16]. Briefly, µ-slides VI 0.1 (ibidi, Martinsried, Germany) were coated with collagen I/III at 300 µg/mL in sodium carbonate/bicarbonate buffer at room temperature (RT) overnight, washed three times with phosphate buffer saline (PBS), blocked with 5% bovine serum albumin for 1 h at RT, and washed with PBS prior to the experiments. Blood samples were obtained using hirudin as an anticoagulant to maintain the platelet function as long as possible to form physiological platelet thrombus. Preliminary experiments determined that 25 µg/mL hirudin was the minimum concentration suitable for the experimental procedures (data not shown). Study subjects had not taken any medication that may have affected platelet function or blood coagulation in the two-week period prior to blood sampling. Standard or pegylated rFVIII (1 IU/mL), rVWF (1.6 IU/mL), pd-VWF/FVIII (calculated as 1.6 IU/mL/1 IU/mL) were added to the whole blood and was perfused in the prepared chamber at the indicated shear rates controlled by a syringe pump NE-1600™ (New Era Pump Systems, Farmingdale, NY). Reactions on the collagen-coated surface under these conditions represented thrombus formation at high shear flow.

***Immunostaining*—**After perfusion, thrombi were fixed with Cytoperm/Cytofix^®^ prior to immunofluorescent staining [14–16]. The fixed thrombi were permeabilized with PBS containing 1% Triton X-100 for 10 min at RT and blocked with serum free protein block (DAKO) for 20 min, followed by incubation overnight at 4 °C with phalloidin-Alexa488 (6 µg/mL), anti-VWF antibody (10 µg/mL) labeled with Alexa568, and anti-FVIII antibody (1 µg/mL) labeled with Alexa647. The stained thrombi were washed 3 times with PBS and mounted in DAKO-fluorescence mounting medium prior to imaging. Preliminary experiments were performed to confirm sufficient infiltration of the fluorescent antibodies into thrombi. Platelets, VWF, and FVIII were visualized using confocal laser scanning microscopy (FV-1000™, Olympus, Tokyo, Japan). Digital images were obtained at 1 μm intervals to a height of 60 μm from the surface. Surface coverage (SC) was measured by calculating the percentage of the area covered by adhering platelets (labeled with phallodin-Alexa488) based on sliced images at 2 μm from the bottom of the thrombus using ImagePro 6.0™ (Media Cybernetics, Rockville, MD). Thrombus height (TH) was calculated by dividing the total volume of the thrombus by SC. SC and TH were measured as markers of initial thrombus formation and thrombus development, respectively [14–16].

***Total-thrombus formation analysis system (T-TAS)—***A microchip flow chamber system (T-TAS^®^; Fujimori Kogyo, Yokohama, Japan) was utilized to analyze thrombus formation under flow conditions as previously described [17, 18]. Briefly, PL (platelet) chips were pre-coated with type I collagen. Hirudin-treated whole blood (300 µL) was perfused into the chamber at flow rates of 12 µL min^-1^, corresponding to initial wall shear rates of 1,000 s^-1^ (high shear). Platelet thrombus formation was visually monitored using a video-microscope located under the microchip, and pressure inside the chamber was monitored and recorded for 10 min or until occlusion. Times to reach 10 kPa (T_10_) and 60 kPa (T_60_) were measured to determine initial platelet thrombus formation. Areas under the curve (AUC) of the obtained pressure readings were calculated as index of thrombus development.

***Data analyses*—**All data and statistical analysis were performed using Graphpad prism 7.0 (Graphpad software Inc., San Diego, CA). The results are shown as means and SD. The statistical differences between groups were evaluated using one-way ANOVA and Tukey’s multiple comparison test. *P*-values < 0.05 were considered as statistically significant.

## Results

### The effect of rVWF and rFVIII on thrombus formation under high shear flow in whole blood from patient with type 1 VWD

**Fig. 1:**
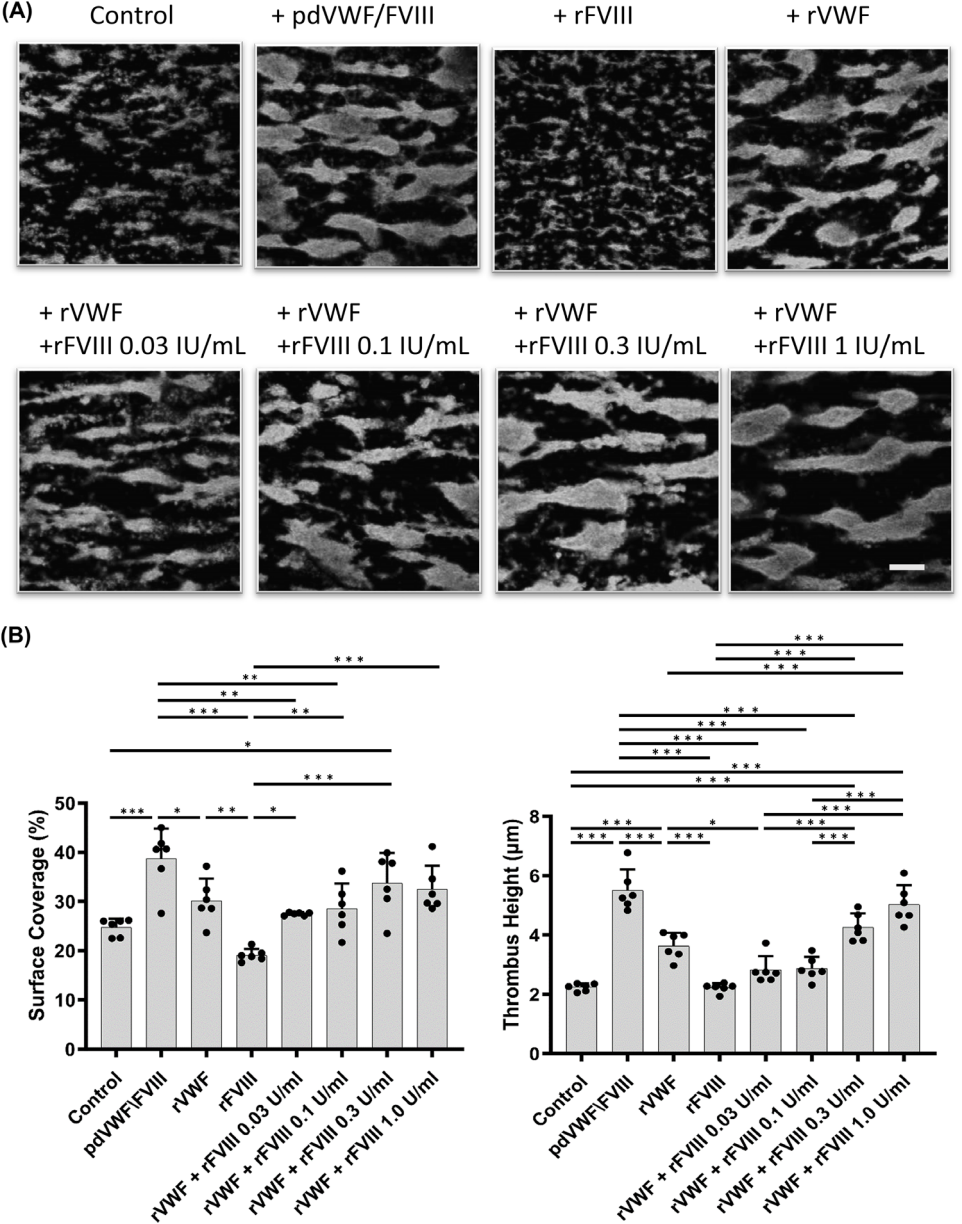
The impact of rVWF and rFVIII on thrombus formation in type 1 VWD whole blood under high shear flow. (*Panel ***A**) rVWF (Vonvendi^®^; 1.6 IU/mL) and/or rFVIII (Advate^®^; 0–1.0 IU/mL), was mixed with whole blood from patients with type 1 VWD, and perfused in the collagen coated perfusion chambers under high shear (2,500 s^− 1^). pd-VWF/FVIII (Confact^®^; VWF:FVIII ratio; 1.6 IU/mL:1.0 IU/mL) and PBS were used as positive or negative controls, respectively. Thrombi were fixed after 4 min perfusion. Platelets were stained with phalloidin-Alexa 488 (6 µg/mL) and were observed using confocal laser scanning microscopy. Representative images are illustrated, and statistical analyses corresponding to the images are shown. The scale bar is set at 30 μm. (*Panel ***B**); Values and error bars represent the mean(± SD) of surface coverage and thrombus height in 15 defined areas (each 133 × 100 μm) randomly selected in 3 separate flow experiments. *p < 0.05, **p < 0.01, ***p < 0.001

Initial experiments were undertaken to examine the ability of rVWF (Vonvendi^®^) or rFVIII (Advate^®^) to promote thrombus formation under high shear flow. rVWF (1.6 IU/mL) and rFVIII (0–1.0 IU/mL) were mixed with whole blood from a patient with severe type 1 VWD and perfused in collagen coated perfusion chambers under high shear flow (Fig. 1A). Prior analysis of the VWD plasma demonstrated VWF ristocetin cofactor activity (VWF:RCo) 5.6%, VWF antigen (VWF:Ag) 7.5%, and FVIII:C 38.0%. PBS was added to the whole blood sample as a negative control and demonstrated that SC and TH were 24.7 ± 1.5% and 2.2 ± 0.11 μm, respectively (Fig. 1B). The positive control, pd-VWF/FVIII (Confact^®^; pd-VWF available in Japan), contained pd-FVIII with a 1.6 to 1 ratio of VWF:RCo to FVIII:C (at that time), and demonstrated SC and TH of 38.7 ± 5.5% and 5.5 ± 0.64 μm, respectively. The addition of rFVIII (1 IU/mL) alone did not augment thrombus formation but rVWF (1.6 IU/mL) significantly enhanced the coagulation reactions (SC: 19.1 ± 1.1% vs. 30.1 ± 4.1%; TH: 2.2 ± 0.14 μm vs. 3.6 ± 0.40 μm). The presence of rFVIII, mixed with rVWF in vitro, increased thrombus formation dose-dependently, and the co-presence of rVWF (1.6 IU/mL) with rFVIII (1.0 IU/mL) (SC 32.5 ± 4.3%, TH 5.0 ± 0.60 μm) was comparable to pd-VWF/FVIII (SC 38.7 ± 5.5%, TH 5.5 ± 0.64 μm). These data suggested that rVWF was similar to pd-VWF, and played an essential role in thrombus formation under high shear flow. The co-presence of rFVIII with rVWF appeared to contribute to TH rather than SC of the formed thrombus.

### Influence of the pre-incubation rVWF and rFVIII in vitro on thrombus formation in type 1 VWD blood

**Fig. 2:**
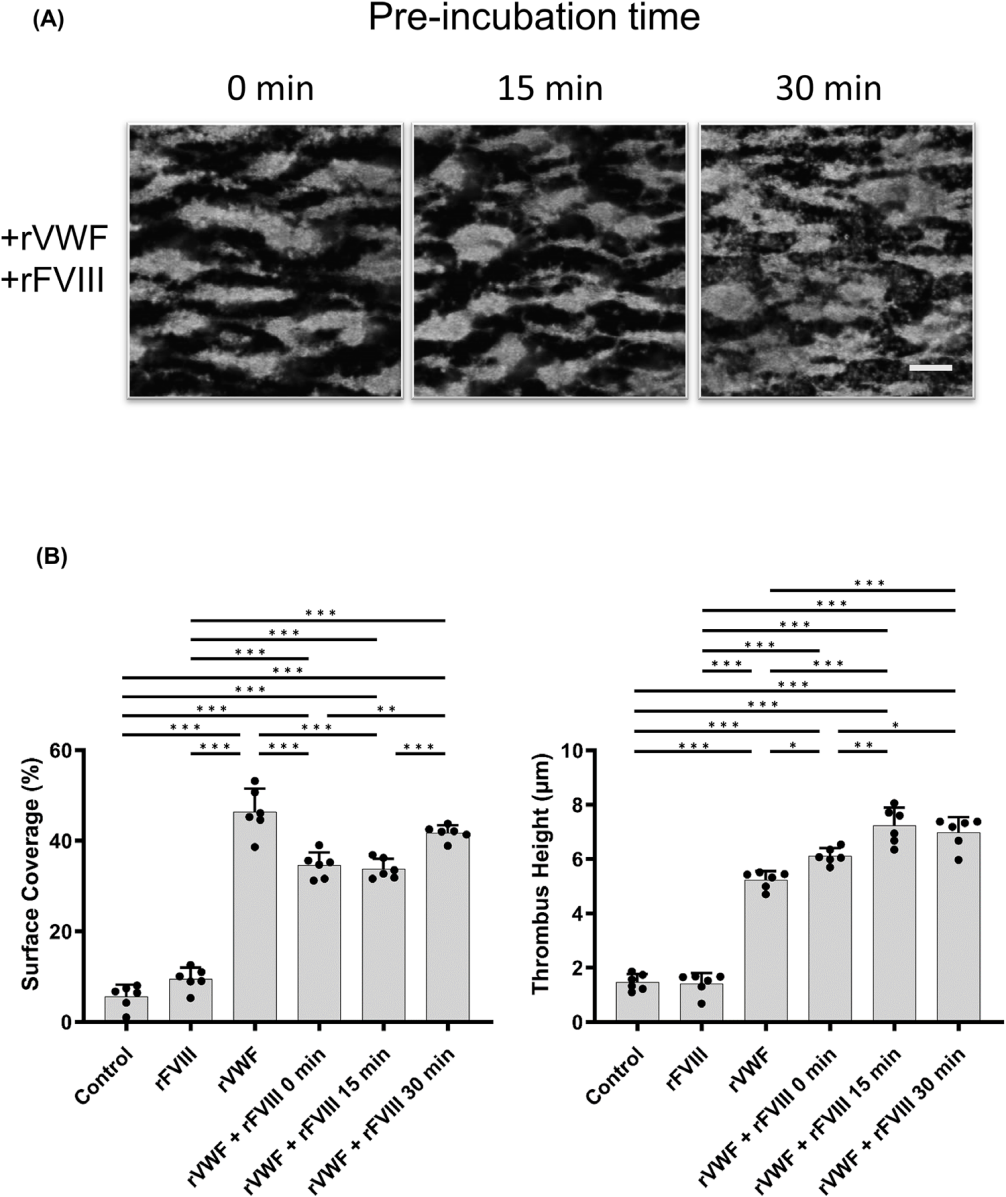
The influence of pre-incubation of rVWF with rFVIII on thrombus formation in type 1 VWD whole blood under high shear flow. (*Panel ***A**) rVWF (1.6 IU/mL) and/or rFVIII (1.0 IU/mL), was incubated with type 1 VWD whole blood for the indicated times (0, 15, and 30 min) and perfused in the collagen coated perfusion chamber under high shear (2,500 s^− 1^). PBS was used as a negative control. Thrombi were fixed after 4 min perfusion. Platelets were stained with phalloidin-Alexa 488 (6 µg/mL) and were observed using confocal laser scanning microscopy. Representative images at the indicated pre-incubation times are illustrated, and statistical analyses corresponding to the images are shown. The scale bar is set at 30 μm. (*Panel ***B**); Values and error bars represent the mean(± SD) of surface coverage and thrombus height in 15 defined areas (each 133 × 100 μm) randomly selected in 3 separate flow experiments. *p < 0.05, **p < 0.01, ***p < 0.001

Pre-incubation of FVIII with rVWF might be necessary to maximize hemostatic effects and could have important clinical implications [19]. Perfusion experiments were performed, therefore, using mixtures of rVWF (1.6 IU/mL) and rFVIII (1 IU/mL) pre-incubated for various times (0, 15, or 30 min; Fig. 2A and B). The results indicated that although the parameters after 30 min incubation were slightly higher than with non-incubated samples (0/15/30 min; SC 34.6 ± 2.6/33.8 ± 2.0/41.7 ± 1.5%, TH 6.1 ± 0.27/7.2 ± 0.61/7.0 ± 0.51 μm, respectively), pre-incubation appeared unlikely to influence the potential for thrombus formation remarkedly. Subsequent experiments with mixtures of rVWF and FVIII products were performed, therefore, without pre-incubation.

### Impact of the association between rVWF and peg-EHL-rFVIII on thrombus formation in type 1 VWD blood

**Fig. 3:**
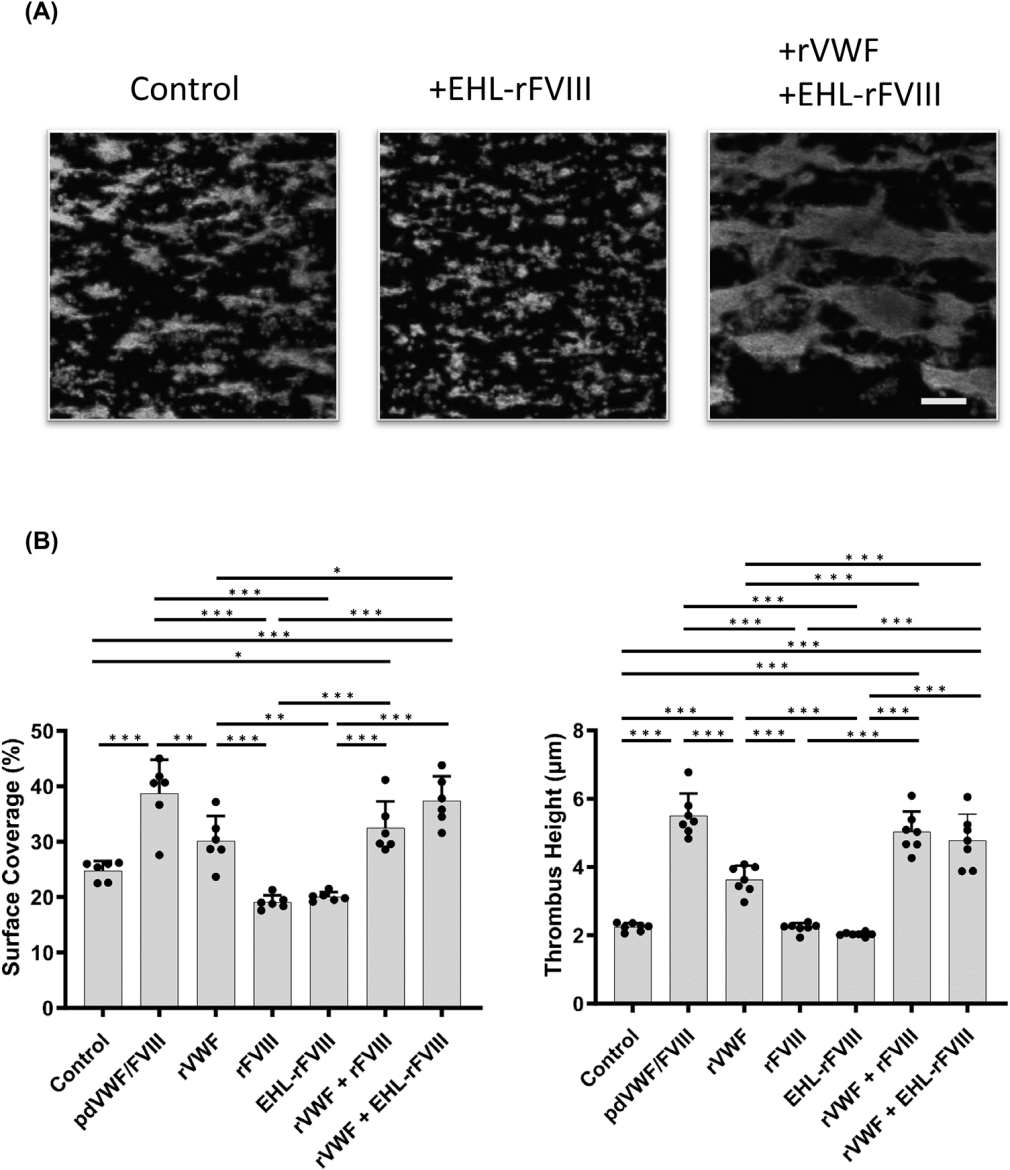
The association between rVWF and peg-EHL-rFVIII in formed thrombi after perfusion at in type 1 VWD whole blood under high shear flow. (*Panel ***A**) rVWF (1.6 IU/mL), rFVIII (1.0 IU/mL) and/or peg-EHL-rFVIII (1.0 U/mL) were added to whole blood from patients with type 1 VWD, and perfused in the collagen coated chambers under high shear (2,500 s^− 1^). pd-VWF/FVIII (1.6 IU/mL/1.0 IU/mL) and PBS were used as positive or negative controls, respectively. Thrombi were fixed after 4 min perfusion. Platelets were stained with phalloidin-Alexa 488 (6 µg/mL) and were observed using confocal laser scanning microscopy. Representative images of PBS, peg-EHL-rFVIII, rVWF/peg-EHL-rFVIII are illustrated, and statistical analyses corresponding to the images are shown. The scale bar is set at 30 μm. (*Panel ***B**) Values and error bars represent the mean(± SD) of surface coverage and thrombus height in 15 defined areas (each 133 × 100 μm) randomly selected in 3 separate flow experiments. *p < 0.05, **p < 0.01, ***p < 0.001, EHL-rFVIII; peg-EHL-rFVIII

Peg-EHL-rFVIII (Adynovate^®^, BAX 855), with both rFVIII chains pegylated, potentiates hemostatic function comparable to standard rFVIII [10]. The mechanisms of EHL-rFVIII-mediated thrombus formation under high shear flow remain to be fully investigated, however. The current perfusion experiments were adapted, therefore, to examine thrombogenesis in the presence of rVWF and peg-EHL-rFVIII, (Fig. 3A). Neither Peg-EHL-rFVIII (1.0 IU/mL; SC 20.1 ± 0.74%, TH 2.0 ± 0.06 μm) nor rFVIII (SC 19.1 ± 1.1%, TH 2.2 ± 0.14 μm) alone affected high-shear thrombus formation in VWD blood (control; SC 24.8 ± 1.6%, TH 2.2 ± 0.12 μm) (Fig. 3B). In the co-presence of rVWF (1.6 IU/mL), however, peg-EHL-rFVIII promoted enhanced thrombus formation similar to rFVIII with rVWF (SC 37.4 ± 4.0 and 32.5 ± 4.3%, p > 0.05; TH 4.8 ± 0.78 and 5.0 ± 0.59 μm, p > 0.05, respectively), indicating that pegylation had little influence on thrombus formation.

### Effects of rVWF and peg-EHL-rFVIII on platelet thrombus formation in type 1 VWD whole blood using an alternative analysis system (T-TAS^®^)

**Fig. 4:**
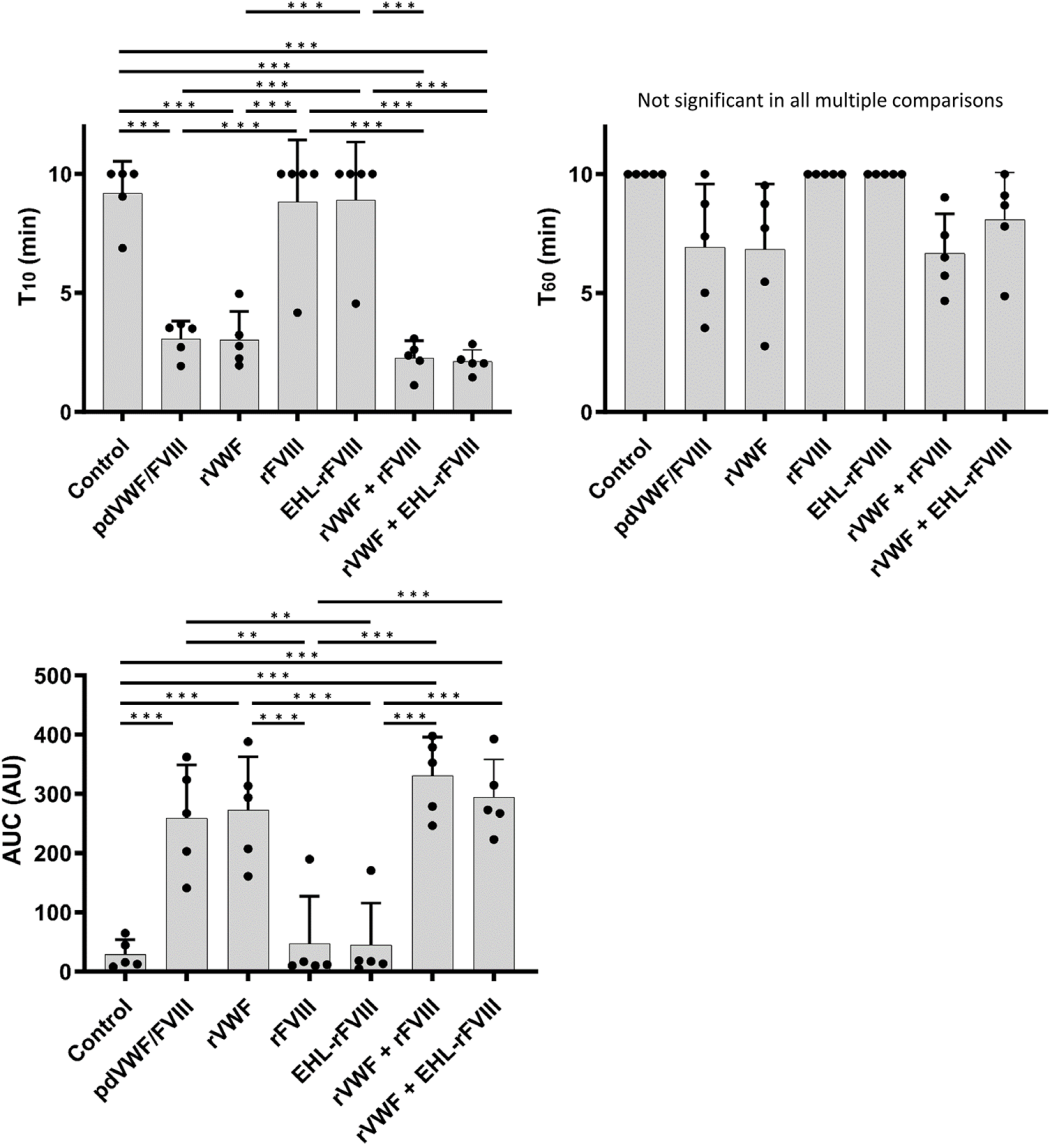
Effects of rVWF and rFVIII or peg-EHL-rFVIII in type 1 VWD whole blood using an alternative flow chamber technique (T-TAS). rVWF (1.6 IU/mL), rFVIII (1.0 IU/mL) and/or Peg-EHL-rFVIII (1.0 U/mL) were added to type 1 VWD whole blood and was perfused at 1,000 s^− 1^ in type I collagen coated flow chambers optimized for T-TAS. pd-VWF/FVIII (1.6 IU/mL/1.0 IU/mL) and PBS was used as positive or negative controls, respectively. The pressure inside the chamber was monitored at 10 and 60 kPa (T_10_ and T_60_) and areas under curve (AUC) were calculated. Measurements of T_10_ or T_60_ at times > 10 min are recorded as 10 min. Values and error bars represent the mean(± SD) of T_10_, T_60_, and AUC in 3 separate flow experiments. *p < 0.05, **p < 0.01, ***p < 0.001, EHL-rFVIII; peg-EHL-rFVIII

The T-TAS^®^ technique uses collagen-coated microchips (PL-chip) to measure VWF-dependent platelet thrombus formation under high shear stress [17]. Our previous studies using this method demonstrated that the potential for platelet thrombus formation was significantly attenuated in VWD blood [18]. In the present study, we confirmed that the parameters of platelet thrombus formation (T_10_, T_60,_ and AUC) in blood from patients with type 1 VWD were reduced at shear rates of 1,000 s^− 1^ (Fig. 4). As expected, the addition of FVIII alone had no effect regardless of the FVIII product type, and rVWF significantly improved platelet thrombus formation, similar to pd-VWF/FVIII in T_10_ and AUC. Also, in keeping with the results discussed above, in the co-presence of rVWF, peg-EHL-rFVIII promoted enhanced thrombus formation to be comparable to that of rVWF with rFVIII.

### Distribution of rVWF and (peg-EHL-)rFVIII in Thrombi formed shear in type 1 VWD blood under high shear flow

**Fig. 5:**
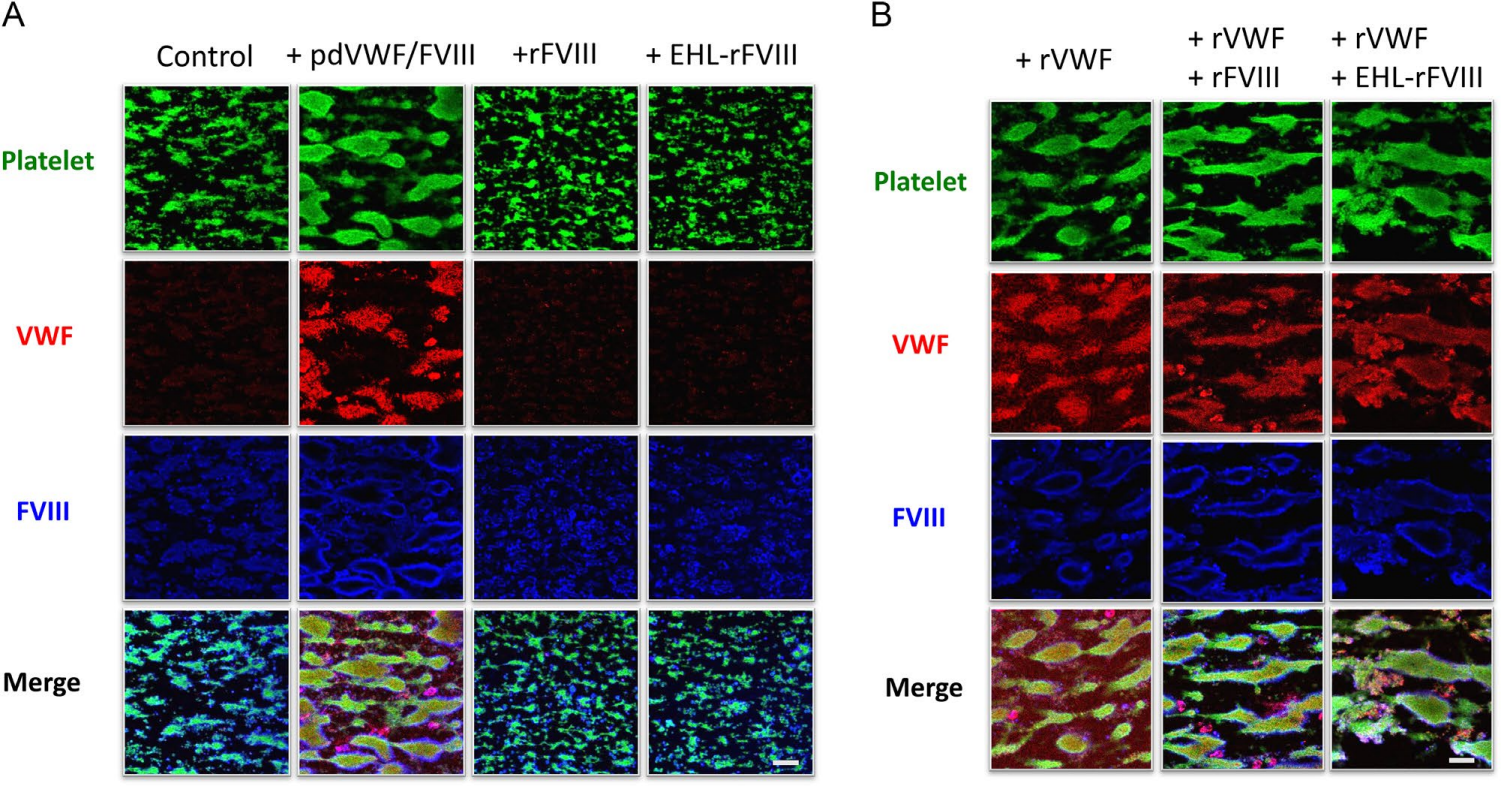
Localization of rVWF, rFVIII, and peg-EHL-rFVIII in formed thrombi in type 1 VWD whole blood at high shear flow. rVWF (1.6 IU/mL), rFVIII (1.0 IU/mL) and/or peg-EHL-rFVIII (1.0 IU/mL) were added to type 1 VWD whole blood from perfused in collagen coated perfusion chamber under high shear (2,500 s^− 1^). pd-VWF/FVIII and PBS (VWF:FVIII = 1.6 IU/mL:1.0 IU/mL) were used as positive or negative controls, respectively. Thrombi were fixed after 4 min perfusion, and stained with phalloidin-Alexa 488 (*green*), anti-VWF antibody-Alexa 567 (*red pseudo-color*), and anti-FVIII antibody-Alexa 647 (*blue pseudo-color*). Representative images are illustrated. Combined color images are defined as merged. The scale bar is set at 30 μm. EHL-rFVIII; peg-EHL-rFVIII

To assess the distribution and relationship between rVWF and (peg-EHL-)rFVIII in formed thrombi under high shear flow, experiments were repeated as described for Fig. 1, and thrombi were examined by immunostaining with phalloidin-Alexa 488 for platelets (*green*), anti-VWF antibody-Alexa 567 for VWF (*red pseudo-color*), and anti-FVIII antibody-Alexa 647 for FVIII (*blue pseudo-color*) (Fig. 5). After the in vitro addition of rFVIII or peg-EHL-rFVIII alone, platelet staining was predominantly observed, and comparable to the negative control (Fig. 5A). In contrast, in the presence of rVWF alone and mixtures of rVWF with rFVIII or peg-EHL-rFVIII (Fig. 5B), staining was evident more heavily throughout the formed thrombi, and both rFVIII products appeared possibly to be co-localized with VWF, as observed in the positive control (pd-VWF/FVIII) illustrated in Fig. 5A, although the core of the thrombi appeared relatively weakly stained for FVIII. The anti-C2 polyAb used in these experiments is known to recognize the platelet (phospholipid)-binding site [11, 12]. It may be, therefore, that platelet binding to FVIII competitively interfered with FVIII-antibody staining, and that the current staining patterns reflected tight interactions between platelets and the FVIII molecule [15]. Nevertheless, these findings indicated that rVWF in the co-presence of peg-EHL-rFVIII or rFVIII contributed extensively to thrombus formation in these circumstances.

## Discussion

Patients with VWD have low plasma levels of VWF and FVIII, and adequate concentrations of both VWF and FVIII have to be maintained during episodes of acute bleeding or surgery [4, 20]. Pd-VWF/FVIII complex concentrates are convenient for replacement therapy [4, 20], but laboratory monitoring and regulation of the hemostatic effectiveness of these individual components is not straightforward. rVWF provides a novel treatment option for patients with VWD without the risk of infection from blood-related pathogens [5, 9]. In a pioneering phase 3 clinical trial in severe VWD, rVWF was initially administered together with rFVIII at a ratio of 1.3 : 1.0 ± 0.2 VWF:RCo/FVIII:C. Bleeding episodes were treated with infusions of 40 to 60 IU/kg VWF:RCo rVWF for minor to moderate bleeds, up to 80 IU/kg VWF:RCo rVWF for major bleeds, and subsequently without rFVIII as long as therapeutic FVIII:C levels were maintained. The results were evaluated using a 4-point scale and demonstrated, 100% hemostatic efficacy rating for all treated bleeding events [8]. Nevertheless, although the treatment was clinically effective, the hemostatic potential of rVWF has not been described in detail.

The present in vitro studies were developed, therefore, to determine the effects of rVWF with rFVIII or peg-EHL-rFVIII on global hemostasis using a perfusion chamber system representing hemodynamic conditions in vivo. Post-perfusion analysis of formed thrombi using immunostaining techniques provided parameters to quantify specific interactions. As we have previously reported [14], SC reflects VWF activity and TH reflects FVIII activity in the presence of VWF. Current data showed that rVWF also increased SC and TH were also increased dose-dependently by rFVIII in the presence of rVWF, and these effects were comparable to pd-VWF/FVIII. Moreover, rFVIII alone had no effect on these parameters, confirming that interactions of rFVIII with rVWF were similar to those with pd-VWF, and were essential for normal hemostasis. It was interesting to note that rVWF, like pdVWF, had a role in localizing FVIII activation to thrombus sites in the bloodstream.

Our findings also demonstrated that in the co-presence of FVIII, rVWF was comparable to pd-VWF in promoting thrombogenesis under high shear, and that although the rVWF contained UL-VWF, excessive thrombus formation was not observed. In our experimental system, rVWF was added to whole blood immediately prior to perfusion, and it may be that thrombus formation was regulated by the proteolysis of the UL-VWF multimers by endogenous ADAMTS13 [21]. These results may be supported by the earlier clinical studies that recorded the initial increase in UL-VWF to 30% after 15 min of (rFVIII/)rVWF administration, followed by a substantial decline [8].

The T-TAS^®^ is an instrument for quantitatively observing thrombus-formation ability in the blood stream based on a new measurement principle, which is used for the analysis and evaluation of platelet thrombus formation (primary hemostatic function) in patients receiving antiplatelet therapy or in patients with congenital platelet dysfunction including VWD [17, 18]. The T-TAS^®^ 01 system using PL chip has been approved by FDA as a medical device (https://www.t-tas.info/news/2020-02-21.html). Even using this clinically relevant measurement method, we were able to confirm that rVWF improved platelet thrombogenicity under high shear as well as pdVWF.

Interactions between VWF and FVIII in the circulation prevent proteolysis of FVIII [19], and pre-incubation of rVWF with rFVIII in vitro might be expected to stabilize FVIII:C prior to administration of the combined therapeutic product in patients with VWD. Our experiments demonstrated, however, that pre-incubation of the mixture was not mandatory to exert its hemostatic potency. The findings were in keeping with the rapid formation of VWF-FVIII complexes, and indicated that straightforward combined infusions of rVWF and rFVIII would be effective without pre-incubation.

Peg-EHL-rFVIII has been shown to have an enhanced survival of FVIII:C in patients with hemophilia A [10]. In addition, products of this type may be used for FVIII replacement therapy in VWD. Limited data are available, however, on comparisons between standard rFVIII and peg-EHL-rFVIII in binding to exogenous rVWF. In this context, therefore, the current perfusion technique demonstrated that there were no significant differences between peg-EHL-rFVIII and standard rFVIII in the analyses of hemodynamic blood coagulation. The localization of rFVIII (Advate^®^) and peg-EHL-rFVIII (Adynovate^®^) was also evaluated using immunostaining. Both types of rFVIII demonstrated similar staining patterns, and although each rFVIII alone was identified in the stained thrombi, neither enhanced thrombus formation. The anti-FVIII antibody used in the present studies reacted with the FVIII C2 domain, and did not identify differences between activated, non-activated or inactivated FVIII. We speculated, however, that the FVIII staining in our experiments reflected the inactivated molecule as previously described [14, 15]. As expected, in the presence of rVWF, both of rFVIII products similarly co-localized with rVWF and increased thrombus formation.

Overall, therefore, our results strengthened the suggestion that hemostatic treatment in patients with VWD using the modified material could be effective with fewer intravenous interventions than with the conventional rFVIII. Pharmacokinetic analyses in the earlier clinical trial demonstrated enhanced stabilization of endogenous and exogenous FVIII in the presence of rVWF compared to the pd-VWF [8], Evaluation of half-life was not possible using our experimental system. It was limitation of this study that only single patient with type 1 VWD participated in the study. Nevertheless, our evidence strongly supports the clinical use of Peg-EHL-rFVIII and rVWF in patients with VWD.

## Conclusion

The effects of high-level rVWF and peg-EHL-rFVIII on thrombus formation were comparable to conventional therapeutic products in a patient’s whole blood with type 1 VWD under high shear flow.

## Data Availability

The datasets used during the current study are available from the corresponding author on reasonable request.

## List of abbreviations

VWF: von Willebrand factor
GP: glycoprotein
FVIII: factor VIII
FVIII:C: factor VIII activity
VWD: von Willebrand disease
pd: plasma-derived
HMW: high molecular weight
rVWF: recombinant VWF product(s)
rFVIII: recombinant FVIII product(s)
UL: ultra-large
ADAMTS13: a disintegrin-like metalloprotease domain with thrombospondin type 1 motifs 13
peg-EHL-rFVIII: pegylated extended-half-life recombinant FVIII product(s)
polyAb: polyclonal antibody
RT: room temperature
PBS: phosphate buffer saline
SC: surface coverage
TH: thrombus height
T-TAS: total-thrombus formation analysis system
PL: platelet
T_10_: time to reach 10 kPa
T_60_: time to reach 60 kPa
AUC: areas under the curve
VWF:RCo: VWF ristocetin cofactor activity
VWF:Ag: VWF antigen
